# Examining colorism, body image, and self-esteem in UK Black and South Asian adolescents

**DOI:** 10.3389/fsoc.2025.1687937

**Published:** 2025-12-02

**Authors:** Nadia Craddock, Aisha Phoenix, Paul White

**Affiliations:** 1 Centre for Appearance Research, School of Social Sciences, University of the West of England, Bristol, United Kingdom; 2 Centre for Public Policy Research, School of Education, Communication and Society, Faculty of Social Science and Public Policy, King’s College London, London, United Kingdom; 3 Department of Mathematics and Statistics, University of the West of England, Bristol, United Kingdom

**Keywords:** colorism, body image, self-esteem, Black adolescents, South Asian adolescents

## Abstract

**Introduction:**

Colorism, a form of appearance-based prejudice in which people are penalized or privileged according to skin shade, hair and facial features, is a pervasive yet underexplored form of bias that affects minoritized ethnic populations. This study examined associations between experiences of colorism and body image and self-esteem among 552 Black and South Asian adolescents (*M*_age_ = 16.1 years) in the United Kingdom.

**Methods:**

Participants completed an online survey that included measures of ingroup and outgroup colorism, internalized colorism, self-reported skin shade, skin shade satisfaction, body image (body esteem), and self-esteem.

**Results:**

On average, Black and South Asian adolescents were not regularly subjected to colorism in their everyday lives. However, both ingroup and outgroup colorism were significantly associated with higher internalized colorism and lower skin shade satisfaction, lower body esteem, and lower self-esteem (all *p* < 0.001). Linear models showed that colorism predicted lower body esteem and lower self-esteem, even after controlling for demographics (e.g., gender, age), self-reported skin shade, and internalized colorism. Ingroup colorism was a stronger predictor of low self-esteem than outgroup colorism though there were no differences between ingroup and outgroup colorism and the relationship with body image. Additionally, analyses showed that internalized colorism mediated the relationship between ingroup colorism and both body image and self-esteem. This mediation pathway was neither observed between outgroup colorism and body image nor self-esteem.

**Discussion:**

Experiencing colourism was associated with worse wellbeing. The relationship between colorism and wellbeing may be explained by different mechanisms based on whether colorism is experienced by one’s own racialized group or not. There is a need for carefully designed culturally responsive interventions.

## 1 Introduction

Colorism refers to the differential treatment people receive the darker their skin is and the further their hair and facial features are from those associated with whiteness (Phoenix and Craddock, 2024a). Rooted in histories of colonialism, slavery, caste stratification, and global white supremacy, colorism intersects with, but is distinct from, racism (Dixon and Telles, 2017). While racism is typically perpetrated by dominant groups against minoritized ethnic populations, colorism occurs both within and between racialized groups (Dixon and Telles, 2017). Extensive research has shown the detrimental impact of racism on adolescent wellbeing (e.g., Priest et al., 2021). However, there has been much less quantitative work exploring the impact of colorism on adolescents (Craddock et al., 2018). This study begins to address this gap by cross-sectionally exploring how experiences of colorism are associated with body image and self-esteem in Black and South Asian adolescents in the United Kingdom (UK). We examine how experiences of colorism may vary according to participants’ gender and racialized group. Additionally, we explore whether the racialization of perpetrators affects how young people respond to colorism by comparing whether findings differ based on experiences of colorism from people from the same racialized minority group (ingroup) or those racialized as White (outgroup).

### 1.1 Colorism and body image

Body image broadly refers to how individuals think and feel about the way their body looks (Cash, 2011). Adolescence is a high-risk development period for experiencing body image concerns. In turn, poor body image during adolescence is associated with worse wellbeing with longitudinal studies showing predictive relationships with later depression, disordered eating, and risky health behaviors (Blundell et al., 2024; Bornioli et al., 2019; Foster et al., 2024). Though body image scholarship has historically focused on weight and shape, increasingly researchers view the term as encompassing other aspects of appearance such as skin shade and complexion (Landor et al., 2024). Importantly, while body image is a psychological construct encompassing individuals’ cognitions, emotions and behaviors about and towards their appearance, it is largely informed by social factors. Sociocultural theories such as the Tripartite Model (Thompson et al., 1999) detail how societal appearance ideals are socially communicated and reinforced by media, family, and peers, which can lead to body dissatisfaction via internalization of these ideals and upwards social comparisons.

The portrayal of light skin as a societal appearance ideal, particularly for women, has been well documented (Landor et al., 2024). In line with the Tripartite Model (Thompson et al., 1999), studies have shown how the media (including social media and advertising), family, and peers promote and reinforce a light-skin ideal. For instance, content analyses have demonstrated how media and advertising perpetuate colorism by prioritizing women with white or light skin to the exclusion of those with dark skin (Hall, 2018; Mitchell, 2020). Scholarship has also shown how social media platforms promote a light-skin ideal via algorithms that exhibit colorist biases, as well as through “beauty” filters that lighten skin (Riccio et al., 2024). Qualitative research has found that family members (parents, siblings, extended family) play a central role in reinforcing colorist appearance ideals, often through casual comments, comparisons, or advice (e.g., *to lighten skin* or *avoid getting darker*) (Mishra et al., 2023; Phoenix and Craddock, 2024a). Peers also perpetuate colorism by bolstering a skin shade hierarchy through appearance-focused comments, teasing, jokes, and romantic preferences (Mathews and Johnson, 2015; Phoenix and Craddock, 2024b). Consistent with the Tripartite Model, these social appearance pressures can harm how individuals think and feel about how they look. This is particularly the case when young people internalize narrow appearance ideals that are unattainable or misaligned with their racialized features and when they make comparisons with those who have lighter skin.

Research focused on experiences of colorism and body image is limited. Qualitative research in the USA and UK has documented how societal appearance ideals that devalue dark skin and natural Black hair textures, shape how Black adolescent girls and young women perceive themselves (Abrams et al., 2020; Gadson and Lewis, 2022; Mathews and Johnson, 2015; Phoenix and Craddock, 2024b). Qualitative work in the UK has also described how South Asian women and adolescent girls experience colorist pressures, particularly from media and family members, and how these influences negatively impact how they think and feel about the way they look (Lewis et al., 2025; Mishra et al., 2023). Turning to quantitative studies, there seems to be even less research that has examined the topic of colorism and body image, particularly among adolescents. In one cross-sectional study with a sample of minoritized ethnic adults in the UK, Craddock et al. (2023a) found that experiences of colorism were indirectly associated with worse body image. Therefore, an aim of this study was to examine whether experiences of colorism are associated with worse body image in minoritized ethnic adolescents living in the UK.

### 1.2 Colorism and self-esteem

Self-esteem can be defined as one’s overall sense of worth and personal value, encompassing perceptions of competence, social acceptance, resilience, and emotional wellbeing (Rosenberg, 1965). A meta-analysis on longitudinal studies showed that self-esteem increases in early and middle childhood, remains constant (but does not decline) in adolescence, and increases strongly in young adulthood. This trend was observed regardless of gender, ethnicity, and country (Orth et al., 2018). Additional meta-analyses have shown that self-esteem predicts depression (Sowislo and Orth, 2013) and social relationships (Harris and Orth, 2020) across the life span, including during adolescence.

Colorist stereotypes may harm the self-esteem of minoritized ethnic adolescents with dark skin. In a longitudinal study following 124 Black adolescent girls, Adams et al. (2020) found that in early adolescence, Black girls with light skin reported higher self-esteem than those with dark skin. Interestingly, this gap narrowed as the girls progressed through high school, as girls with medium and dark skin reported increases in self-esteem over time. However, this study did not measure colorism, so it is hard to draw clear conclusions on the role of colorism and self-esteem for these young women. Several cross-sectional studies with adults have shown a relationship between experiences of colorism and self-esteem. For instance, in a study with women in Pakistan aged 18–40, those who reported more experiences of colorism were also more likely to report lower self-esteem (Sharif and Siddique, 2020). Similarly, in a UK sample of minoritized ethnic adults, more frequent experiences of colorism from people belonging to the same racialized group were associated with lower self-esteem (Craddock et al., 2023b). Finally, in a study with Latinx adolescents living in the USA, those who reported experiencing more colorism were more likely to report lower self-esteem and greater depressive symptoms (Centeno et al., 2023).

### 1.3 Ingroup and outgroup colorism

An important feature of colorism is that it can operate within and between racialized groups, such that individuals can experience colorism from the same racialized group (ingroup) and from other racialized groups (outgroup). Studies in the USA and UK have started to explore how ingroup and outgroup colorism affect wellbeing, focusing on the dominant racialized group (White people) as the outgroup in both contexts. These studies indicate that ingroup colorism is often more strongly associated with worse wellbeing outcomes than outgroup colorism (Craddock et al., 2023b; Monk, 2021; Oh et al., 2021). For instance, Monk (2021) showed that among Black American adults, experiences of colorism from other Black people were significantly associated with worse physical and mental health outcomes, over and above other forms of discrimination. Similarly, Oh et al. (2021) found that ingroup colorism was more strongly linked to elevated risk for anxiety and other psychiatric disorders in Black adults in the USA than outgroup colorism. Finally, in a sample of minoritized ethnic people in the UK, Craddock et al. (2023b) found that ingroup colorism was associated with lower self-esteem and perceived social support, while outgroup colorism was associated with greater anxiety.

As adolescence is marked by heightened sensitivity to peer and social evaluation, it is possible that ingroup colorism may be particularly harmful due to its occurrence within presumed safe or affirming spaces such as families, schools, and religious communities (James et al., 2018). Indeed, qualitative studies with minoritized ethnic adults in the UK document narratives of individuals experiencing colorism from family members during childhood which had long-term effects on their skin shade satisfaction, body image, and self-worth (Mishra et al., 2023; Phoenix and Craddock, 2024a). Additionally, qualitative research has also shown how being overlooked as romantic partners by men racialized in the same way can lead to feelings of rejection by, and tensions between, minoritized ethnic women, which can impact how these women see themselves (Phoenix and Craddock, 2024b). This may be even more potent as adolescents start navigating romantic relationships. In contrast, outgroup colorism, though still harmful, may be interpreted through existing frameworks of racism or intergroup conflict and so individuals may be able to distance themselves from the prejudice they encounter.

### 1.4 Skin shade satisfaction, body image, and self esteem

Existing research with Black adolescents has shown that skin shade satisfaction is associated with more general body satisfaction or body esteem. In a sample of 252 Black adolescents in the USA, skin shade satisfaction was significantly associated with higher appearance esteem, even when controlling for weight and muscle tone satisfaction (Ladd et al., 2022). Notably, these associations were similar for adolescent girls and boys (Ladd et al., 2022). In a prospective study following 1,213 Black adolescent girls, Parker et al. (2022) found that skin shade satisfaction predicted later body satisfaction. In fact, Parker et al. (2022) observed that when participants were dissatisfied with their skin shade, they were at greater risk of disordered eating – a relationship mediated by body (dis)satisfaction. In a second longitudinal study following Black women from adolescence to the age of 40, Parker et al. (2024) reported that greater skin shade satisfaction at ages 12 and 15 was indirectly associated with greater body satisfaction in adulthood, with body satisfaction at age 19 serving as a mediator. Parker et al. (2024) also reported that skin shade satisfaction at age 12 directly predicts greater self-esteem in adulthood.

### 1.5 The present study

This study aimed to explore whether experiences of colorism were cross-sectionally associated with body image and self-esteem in two minoritized ethnic groups of adolescents living in the UK: Black and South Asian 13–19-year-olds. According to the 2021 Census, (Office for National Statistics, 2022) 9.3% of the population of England and Wales identified as Asian, Asian British or Asian Welsh (including Indian, Pakistani, Bangladeshi and other Asian) and 4.0% as Black, Black British, Caribbean or African groups. In Scotland’s 2022 Census, (National Statistics, 2024) those identifying as Asian, Asian Scottish or Asian British accounted for 3.9% of the total population and those identifying as Black, Black Scottish, or Black British made up 1.2% of the population in Scotland. By focusing on Black and South Asian adolescents in the UK, relatively under-studied populations in colorism research, we aim to contribute to the growing body of scholarship examining how intersecting identities and social contexts shape youth wellbeing.

Research Question (RQ) 1: *Are experiences of colorism associated with worse body image when controlling for self-reported skin shade, internalized colorism, and skin shade satisfaction?*

We hypothesized that experiences of colorism would be negatively associated with body image, even when controlling for self-reported skin shade, satisfaction with one’s skin shade, and internalized colorist beliefs.

RQ2: *Are experiences of colorism associated with lower self-esteem when controlling for self-reported skin shade, skin shade satisfaction, and internalized colorism?*

We hypothesized that experiences of colorism would be negatively associated with self-esteem, even when controlling for self-reported skin shade, satisfaction with one’s skin shade, and internalized colorist beliefs.

RQ3: *Are there differences in the strength of association for ingroup versus outgroup colorism?*

Consistent with studies focused on adults, we anticipated that ingroup colorism would have a stronger relationship with negative body image and self-esteem than outgroup colorism.

RQ4: *Does internalized colorism mediate the relationship between both ingroup and outgroup colorism with the two psychological outcome variables: body image and self-esteem?*

We expected that the degree to which individuals internalized colorist ideas would help explain the following four relationships: (i) ingroup colorism and body image, (ii) ingroup colorism and self-esteem, (iii) outgroup colorism and body image, and (iv) outgroup colorism and self-esteem.

Finally, further to the above hypotheses, we also explored some additional questions looking at the differences in experience by gender and racialized group.

## 2 Method

### 2.1 Participants

Participants were 552 young people living in the UK aged between 13 and 19 (*M*_age_ = 16.1 years) who identified as Black or South Asian. Most participants (86.4%) were in full-time education. Among the remainder, 7.1% had completed formal education, 3.8% were in part-time education, 2.0% were taking a break from education, and 0.7% preferred not to share. Most participants (84.4%) had two parents/guardians and almost three-quarters of participants (72.7%) had at least one parent who had been to university. For further participant characteristics, see Table 1.

**Table 1:** Participant characteristics (*N* = 552).

Characteristic	*N*	%
Gender		
Girl/Woman	294	53.3%
Boy/Man	254	46.0%
Non-Binary	3	0.5%
*Prefer not to say*	1	0.2%
Racialized Group		
Black	301	54.5%
South Asian	251	45.5%
Born in the UK		
Yes	505	91.5%
No	47	8.5%
*Prefer not to say*	0	0
Skin Shade (compared with ingroup)		
Very Dark	8	1.4
Dark	143	25.9
Medium	266	48.2
Light	114	20.7
Very Light	16	2.9
*Prefer not to say*	5	0.9
Sexual orientation		
Heterosexual	488	88.4
Gay/Lesbian	8	1.4
Bisexual/Pansexual	24	4.3
Asexual	20	3.6
Questioning/Other	2	0.4
*Prefer not to say*	10	1.8
Religious beliefs		
Christian	270	48.9
Hindu	54	9.8
Muslim	168	30.4
Other (e.g., Sikh)	14	2.5
No religion	41	7.4
*Prefer not to say*	5	0.9
UK region		
London	232	42.0
North (North West, North East)	65	11.8
South (South West, South East)	45	8.2
Midlands or East of England	136	24.6
Yorkshire	49	8.9
Northern Ireland, Scotland or Wales	25	4.5

### 2.2 Measures

#### 2.2.1 Skin shade

**Figure 1:**
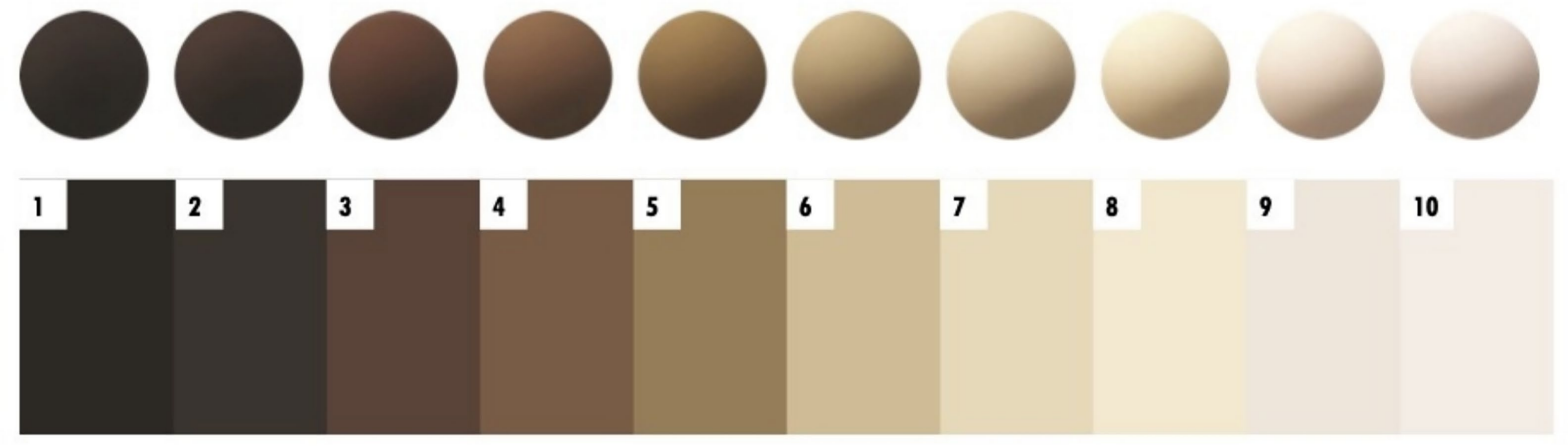
Monk’s (2021) skin tone chart.

Participants were asked to complete two self-reported skin shade measures. First, participants were asked to indicate whether, compared with their racialized group peers, they had ‘very dark’, ‘dark’, ‘medium’, ‘light’, or ‘very light’ skin. Second, participants were asked to select the color that best resembled their skin shade on Monk’s 2021 Skin Tone Chart. Participants were shown both the 10 orbs and swatches (Figure 1). The darkest skin shade was 1 and the lightest was 10.

#### 2.2.2 Internalized colorism

Internalization of colorism was assessed using the Ingroup Colorism Scale (ICS; Harvey et al., 2017). This scale comprises 20 items and five subscales: self-concept, affiliation, attraction, impression formation, and upward mobility. Small modifications were made to make items more appropriate for an ethnically diverse UK sample (e.g., “Black Americans” was replaced with “People of Color”) and for consistency of terms across the survey to avoid confusion (e.g., “skin tone” was replaced with “skin shade”). Sample items include “Skin shade affects how much money you can make” and “Most of my friends tend to be the same skin shade.” Participants provided responses on a 7-point Likert-type scale (1 = *Strongly Disagree*, 7 = *Strongly Agree*). A global score was calculated by summing and averaging all 20 items with higher scores indicating higher internalized colorism. The overall Cronbach’s alpha was 0.88 (0.86 for girls, 0.90 for boys; 0.88 for Black adolescents, 0.89 for South Asian adolescents).

#### 2.2.3 Everyday colorism

Experiences of colorism were captured by the recently validated Everyday Colorism Scale for Adolescents (ECS-A; Craddock et al., in press), adapted from the adult version of the scale (Craddock et al., 2023b). The ECS-A scale has 16 items and participants are asked to indicate the extent to which they have experienced a particular colorism scenario on a 5-item Likert-type scale (1 = *Never*, 5 = *Always*). Participants were asked to respond twice, based on experiences of colorism from their ingroup (i.e., other Black or South Asian people depending on the participant’s racialization) and the majority outgroup (White people). Participants were given the following guidance before answering: *“You may wish to think of people who are family members, friends or peers, teachers/school staff, members of the public, shopkeepers, police/security guards etc. when responding to these statements. This means you may think of other young people as well as adults.”* Overall Ingroup Cronbach’s alpha was 0.94 (0.94 for girls, 0.95 for boys; 0.94 for Black adolescents, 0.95 for South Asian adolescents). Overall Outgroup Cronbach’s alpha was 0.96 (0.96 for girls, 0.97 for boys; 0.96 for Black adolescents, 0.97 for South Asian adolescents).

#### 2.2.4 Skin shade satisfaction

Skin shade satisfaction was measured with a single item: how satisfied are you with your skin shade (how dark or light your skin color is)? Responses were provided on a 5-item Likert-type scale (1 = *Very Dissatisfied*, 5 = *Very Satisfied*).

#### 2.2.5 Body image

The 10-item Appearance Evaluation subscale from the Body Esteem Scale for Adults and Adolescents (BESAA; Mendelson et al., 2001) was used to measure participants’ body image. Specifically, this subscale assesses overall self-appraisals of one’s own appearance (*“I like what I see when I look in the mirror”*). Items are rated on a 5-point Likert scale (1 = “*Neve*r” to 5 = “*Always*”). A mean total score was calculated, with lower scores indicating worse body image. The BESAA has demonstrated high internal consistency and reliability and has been validated for use with adolescents as well as adults (Mendelson et al., 2001). It has also been used previously in studies of Black adolescents in the U.S. (Ladd et al., 2022). Cronbach’s alpha for the full sample was 0.93 (0.94 for girls, 0.89 for boys; 0.93 for Black adolescents, 0.92 for South Asian adolescents).

#### 2.2.6 Self-esteem

Rosenberg’s Self-Esteem Scale (RSES; Rosenberg, 1965) was used to measure self-esteem. The scale contains 10 items, with the response options ranging from 1 (*Strongly Disagree*) to 4 (*Strongly Agree*). A sample item is “on the whole, I am satisfied with myself.” Five items were reverse scored so that higher global mean scores indicate higher self-esteem. Overall Cronbach’s alpha was 0.84 (0.87 for girls, 0.79 for boys; 0.82 for Black adolescents, 0.84 for South Asian adolescents).

### 2.3 Procedure

Ethical approval was granted by King’s College London’s Social Science, Humanities and Law Research Ethics Sub-Committee (RESCM-23/24–34003) and data were collected from July to September 2024. The research agency Childwise recruited participants, advertising the study on partner research panels. Participants had to meet the following eligibility criteria: (i) be aged 13–19 years, (ii) be Black or South Asian (and not of mixed racialized backgrounds), (iii) live in the UK, and (iv) be born in the UK or have moved to the UK before starting primary school. Anyone not meeting these criteria was excluded from the study.

For participants aged 13–16, parental consent was obtained before the child provided their consent to participate in the study. For participants aged 17–19, parental consent was not required (in line with KCL ethics committee guidelines), so after reading the information sheet, individuals were invited to provide their informed consent prior to completing the survey. The survey was regularly monitored by the research agency for any safeguarding disclosures in the open-ended response boxes. Anonymized data was sent to the study authors for analysis. Participants were allocated points via the research panel to which they or their parent was subscribed. The points could be converted to shopping vouchers.

### 2.4 Data analysis

All analyses were conducted on SPSS v 29. The data is amenable to analysis using established statistical techniques (Pearson-Bravais correlation coefficient, independent samples t-test, multivariable linear models, and mediation analysis). To reduce Type 1 error with multiple comparisons, significance was set at *p* < 0.01.

Correlation analyses and between group comparisons are presented as exploratory analyses to better understand the gender differences within the two racialized groups and to explore nuanced correlational effects.

Multivariable linear models were used to test study hypotheses. The variables included in the models (in-group and out-group experiences of colorism, self-reported skin shade, internalized colorism, skin shade satisfaction, age, racialized group, and gender) were pre-specified without reliance on algorithmic variable selection. Model assumptions were examined with no marked deviation from expectation.

Building on the linear models, four mediation models were tested using PROCESS Model 4 (Hayes, 2022) to examine whether internalized colorism mediates the relationship between both ingroup and outgroup colorism and the two psychological outcomes (body esteem and self-esteem). The same covariates used in the linear models were controlled for. Statistical significance was judged using the bootstrap (5,000 bootstrap samples).

## 3 Results

### 3.1 Descriptive statistics

Table 2 provides the correlations and descriptive information for key study variables. Mean values for ingroup and outgroup colorism were low, equivalent to only ‘rarely’ experiencing colorism in their everyday lives. Almost one in five young people (17.8%) reported never experiencing ingroup colorism, while 10.3% reported never experiencing colorism from White people. However, almost a third of participants (30.8%) provided a mean score of 2 or higher for ingroup colorism and more than half of participants (55.3%) gave a mean score of 2 or higher for outgroup colorism, indicating the relevance of the prejudice for a sizeable proportion of the study population.

**Table 2:** Correlation matrix (*N* = 552).

Variable	1	2	3	4	5	6	7	8	9
1. ECS-A In	–								
2. ECS-A W	0.578**	–							
3. ICS	0.259**	0.239**	–						
4. RSES	−0.379**	−0.280**	−0.203**	–					
5. BESAA-AE	−0.332**	−0.275**	−0.178**	0.741**	–				
6. Skin Shade Sat	−0.252**	−0.171**	−0.110	0.381**	0.439**	**–**			
7. Shade Comparison	−0.080	−0.110	−0.021	−0.058	−0.036	0.072	–		
8. Shade Chart	−0.104	−0.205**	−0.035	0.030	0.058	0.024	−0.701**	–	
9. Age	0.155**	0.115*	0.001	−0.326**	−0.303**	−0.112*	−0.027	−0.010	**–**
Descriptive statistics									
*N*	538	533	520	498	536	552	547	549	552
Mean	1.76	2.28	3.38	3.11	3.70	4.27	3.02	5.08	16.1
SD	0.75	0.97	1.02	0.56	0.96	0.87	0.80	1.63	2.08
Possible Range	1–5	1–5	1–7	1–4	1–5	1–5	1–5	1–10	13–19

*p < .01, **p < .001. ECS-A In – ingroup colorism; ECS-A W – colorism from white people, ICS – internalized colorism, RSES – self-esteem, BESAA-AE – body esteem, appearance evaluation, Skin Shade Sat – skin shade satisfaction.

The two skin shade measures were highly correlated (*r* = −0.701) such that participants who indicated they had comparatively darker skin were more likely to select a darker skin shade. Black participants were most likely to rate their skin shade as ‘dark’ (41%), closely followed by ‘medium’ skin (38%) relative to other Black people. South Asian participants were most likely to report having ‘medium’ (60%) skin when thinking about other South Asian people. The median skin shade was 4 for Black participants and 6 for South Asian participants (two shades lighter than Black participants). Notably, most adolescents (85.0%) indicated that they were satisfied or very satisfied with their skin shade, giving an average score of 4.27 where 4 = satisfied and 5 = very satisfied. A total of 9.4% of adolescents reported they were neither satisfied nor dissatisfied, 5.1% reported they were dissatisfied and 0.5% selected very dissatisfied.

Experiences of both ingroup and outgroup colorism were significantly associated with higher internalized colorism, lower skin shade satisfaction, lower body esteem, and lower self-esteem (*p* < .001). There was also a significant correlation between both ingroup and outgroup colorism with age, such that older adolescents reported more frequent experiences of colorism from both their racialized minority peers and White people.

Table 3 shows some differences in means by gender within racialized groups. Of note, among Black participants, girls have significantly lower mean internalized colorism (*p* = .002), lower self-esteem (*p* = .003), lower body esteem (*p* < .001) and lower skin shade satisfaction (*p* < .001) scores than boys. There were no significant differences in study variables between South Asian girls and boys at the threshold of *p* < .01, aside from age, where girls were significantly older than boys in this sample.

**Table 3:** Means, independent sample *t*-tests and effect size (Cohen’s *d*) comparing the scores of adolescent girls with boys within each racialized group.

Black participants						
Variable	Girls (*n* = 163)	Boys (*n* = 136)	*t*	*df*	*p*	*d*
ECS-A In	1.86	1.67	2.154	289	.016	0.254
ECS-A W	2.50	2.36	1.257	288	.105	0.148
ICS	3.28	3.62	−2.934	287	.002	0.347
RSES	3.16	3.33	−2.770	265	.003	0.340
BESAA-AE	3.71	4.19	−4.610	291	<.001	0.541
Skin Shade Sat	4.30	4.65	−3.880	298	<.001	0.450
Skin Shade Com	2.70	2.80	−1.024	298	.153	0.120
Skin Shade Chart	4.23	4.73	−2.764	297	.003	0.321
Age	16.24	15.66	2.414	298	.008	0.280

South Asian participants						
Variable	Girls (*n* = 131)	Boys (*n* = 117)	*t*	*df*	*P*	*d*
ECS-A In	1.83	1.62	2.205	241	.028	0.283
ECS-A W	2.09	2.05	0.286	238	.775	0.037
ICS	3.20	3.48	−2.051	226	.041	0.272
RSES	2.90	3.06	−2.128	225	.034	0.283
BESAA-AE	3.34	3.55	−1.688	237	.093	0.219
Skin Shade Sat	3.97	4.14	−1.452	246	.148	0.185
Skin Shade Com	3.27	3.21	0.813	245	.417	0.104
Skin Shade Chart	5.98	5.65	1.974	244	.049	0.252
Age	16.84	15.69	4.608	246	<.001	0.586

Two-sided, equal variance assumed.

Table 4 shows some differences in means by racialized group within gender. Compared with South Asian girls, Black girls reported significantly more frequent experiences of colorism from White people, yet indicated higher self-esteem, higher body esteem, and higher skin shade satisfaction. Black girls also reported having darker skin compared with other Black people more frequently than South Asian girls reported having darker skin compared with other South Asians. Black girls also selected a darker skin shade on the skin shade chart than South Asian girls. The same pattern of results was observed comparing Black and South Asian boys. The only exception was that the difference in scores for colorism from White people did not meet the criterion of *p* < .01.

**Table 4:** Means, independent sample *t*-tests and effect size (Cohen’s *d*) comparing the scores of Black and South Asian adolescents within girls and boys.

Girls						
Variable	Black (*n* = 163)	South Asian (*n* = 131)	*t*	*df*	*p*	*d*
ECS-A In	1.86	1.83	0.359	286	.720	0.043
ECS-A W	2.50	2.09	3.686	279	<.001	0.442
ICS	3.28	3.20	0.715	277	.238	0.086
RSES	3.16	2.90	3.598	259	<.001	0.448
BESAA-AE	3.71	3.34	3.065	286	<.001	0.363
Skin Shade Sat	4.30	3.97	3.138	292	<.001	0.368
Skin Shade Com	2.70	3.27	−6.704	291	<.001	0.788
Skin Shade Chart	4.23	5.98	−11.092	292	<.001	1.302
Age	16.24	16.84	−2.549	292	.006	0.293

Boys						
Variable	Black (*n* = 137)	South Asian (*n* = 117)	*t*	*df*	*P*	*d*
ECS-A In	1.67	1.62	0.567	244	.565	0.074
ECS-A W	2.36	2.05	2.483	247	.014	0.316
ICS	3.62	3.48	0.999	236	.319	0.130
RSES	3.33	3.06	4.126	231	<.001	0.542
BESAA-AE	4.19	3.55	6.257	242	<.001	0.805
Skin Shade Sat	4.65	4.14	5.371	252	<.001	0.676
Skin Shade Com	2.80	3.21	−4.027	248	<.001	0.511
Skin Shade Chart	4.73	5.65	−4.642	249	<.001	0.588
Age	15.66	15.69	−0.140	252	<.444	0.018

Two-sided, equal variance assumed.

Looking at correlations by racialized group (Table 5), ingroup colorism was positively associated with outgroup colorism and internalized colorism and negatively associated with self-esteem, body esteem and skin shade satisfaction for both Black and South Asian adolescents (all *p* < 0.001). Similarly, outgroup colorism was positively associated with internalized colorism and negatively associated with self-esteem, body esteem and skin shade satisfaction for both Black and South Asian adolescents (all *p* < .001).

**Table 5:** Correlation matrix split by racialized group (Black adolescents – top triangle, South Asian adolescents – bottom triangle).

Variable	1	2	3	4	5	6
1. ECS-A In	–	0.567**	0.244**	−0.403**	−0.350**	−0.231**
2. ECS-A W	0.606**	–	0.257**	−0.322**	−0.309**	−0.226**
3. ICS	0.275**	0.206*	–	−0.264**	−0.262**	−0.151
4. RSES	−0.404**	−0.380**	−0.176	–	0.718**	0.393**
5. BESAA-AE	−0.352**	−0.377**	−0.113	0.729**	–	0.413**
6. Skin Shade Sat	−0.306**	−0.224**	−0.102	0.298**	0.389**	**–**

*p < .01, **p < .001.

Looking at correlations by gender (Table 6) for both adolescent girls and boys, ingroup colorism was significantly associated with higher outgroup colorism and internalized colorism scores and lower self-esteem, body esteem, and skin shade satisfaction scores. There were no other significant associations for ingroup colorism at the threshold of *p* < .01. Regarding outgroup colorism, for both adolescent girls and boys, colorism from White people was significantly associated with higher internalized colorism and lower self-esteem. For adolescent girls, but not adolescent boys, colorism from White people was also associated with lower body esteem and lower skin shade satisfaction.

**Table 6:** Correlation matrix split by gender (girls top triangle, boys bottom triangle).

Variable	1	2	3	4	5	6
1. ECS-A In	–	0.563**	0.211**	−0.412**	−0.318**	−0.228**
2. ECS-A W	0.594**	–	0.226**	−0.343**	−0.365**	−0.203**
3. ICS	0.379**	0.280**	–	−0.221**	−0.147	−0.136
4. RSES	−0.307**	−0.183*	−0.256**	–	0.767**	0.367**
5. BESAA-AE	−0.316**	−0.136	−0.312**	0.678**	–	0.394**
6. Skin Shade Sat	−0.241**	−0.110	−0.147	0.363**	0.469**	**–**

*p < .01, **p < .001.

Table 7 summarizes correlations between skin shade satisfaction and measures by gender and racialized group. Skin shade satisfaction is consistently associated with both self-esteem (*p* < .001) and body esteem (*p* < .001) for all gender and racialized groups. Skin shade satisfaction is negatively correlated with ingroup colorism (*p* < .01), except this correlation does not achieve the more stringent levels of statistical significance of *p* < .01 for Black Girls. Similarly, skin shade satisfaction is negatively correlated with colorism from White people (*p* < .01), except this correlation does not achieve the more stringent levels of statistical significance of *p* < .01 for South Asian Boys. Skin shade satisfaction is negatively correlated with internalized colorism (*p* < .01) only for Black Girls, and skin shade satisfaction is significantly correlated with self-reported skin shade (*p* < .01) and self-reported within group skin shade comparison (*p* < .01) only for Black Boys. That is, for Black girls and for South Asian boys and girls, there was no significant relationship between the two measures of self-reported skin shade and skin shade satisfaction. Interestingly, for Black boys, those who reported having darker skin reported greater satisfaction with their skin shade. These analyses outline the importance of skin shade satisfaction but with context varying levels of importance.

**Table 7:** Correlations between study variables and skin shade satisfaction.

	Black		South Asian	
Variable	Girls (*n = 163*)	Boys (*n = 134*)	Girls (*n = 131*)	Boys (*n = 116*)
ECS In	−0.195	−0.237*	−0.284*	−0.301*
ECS White	−0.207*	−0.231*	−0.309**	−0.121
ICS	−0.218*	−0.176	−0.070	−0.181
RSES	0.377**	0.353**	0.301**	0.274*
BESAA-AE	0.350**	0.440**	0.399**	0.362**
Skin shade chart	0.121	0.234*	0.165	0.165
Skin shade comparison	0.029	0.242*	0.169	0.139

*p < .01; **p < .001.

### 3.2 Hypothesis testing

As hypothesized (H1), experiences of colorism were significantly associated with worse body image, even when self-reported skin shade, internalized colorism, skin shade satisfaction, and key demographics (age, racialized group, gender) were included in the model, Adjusted *R*^2^ = 0.381, *F*(9, 480) = 33.831, *p* < .001 (see Table 8). Similarly, as hypothesized, experiences of colorism were significantly associated with lower self-esteem, even when self-reported skin shade, internalized colorism, skin shade satisfaction, and key demographics (age, racialized group, gender) were included in the model, Adjusted *R*^2^ = 0.347, *F*(9, 449) = 27.544, *p* < .001 (Table 9).

**Table 8:** All participants, multivariate linear model predicting body esteem, adjusted *R*^2^ = 0.381.

Variable	Standardized coefficients *b*	*t*	*p*	95% CI
Skin shade				
Comparative	0.173	3.364	<.001	0.074, 0.280
Skin tone chart	0.245	4.508	<.001	0.140, 0.358
Skin shade attitudes				
Skin shade satisfaction	0.278	6.997	<.001	0.200, 0.357
Internalised colorism	−0.110	−3.050	.002	−0.191, −0.039
Experiences of colorism				
Ingroup	−0.160	−2.737	.006	−0.218, −0.037
Outgroup – White	−0.124	−2.763	.006	−0.210, −0.031
Demographics				
Girl	−0.105	−2.753	.006	−0.181, −0.030
Black	0.244	5.781	<.001	0.162, 0.331
Age	−0.197	−5.258	<.001	−0.271, −0.124

All variables standardized.

**Table 9:** All participants, multivariate linear model predicting self-esteem, adjusted *R*^2^ = 0.347.

Variable	Standardized coefficients *b*	*t*	*p*	95% CI
Skin shade				
Comparative	0.176	3.201	<.001	0.070, 0.289
Skin tone chart	0.200	3.462	<.001	0.088, 0.316
Skin shade attitudes				
Skin Shade Satisfaction	0.212	5.040	<.001	0.130, 0.300
Internalized colorism	−0.116	−2.842	.005	−0.189, −0.031
Experiences of colorism				
Ingroup	−0.220	−4.603	<.001	−0.315, −0.127
Outgroup (White)	−0.094	−1.975	.049	−0.180, 0.000
Demographics				
Girl	−0.075	−1.885	.060	−0.153, 0.005
Black	0.210	4.665	<.001	0.123, 0.301
Age	−0.206	−5.213	<.001	−0.283, −0.127

All variables standardized.

We found partial support for our third hypothesis (H3) that ingroup colorism would have a stronger association with both lower body esteem and self-esteem. Contrary to our expectations, we found that ingroup and outgroup colorism contributed similar variance in the model for body esteem. However, in the model for self-esteem, in line with our hypothesis, ingroup colorism contributed more strongly than outgroup colorism, though both remained significant predictors.

**Figure 2:**
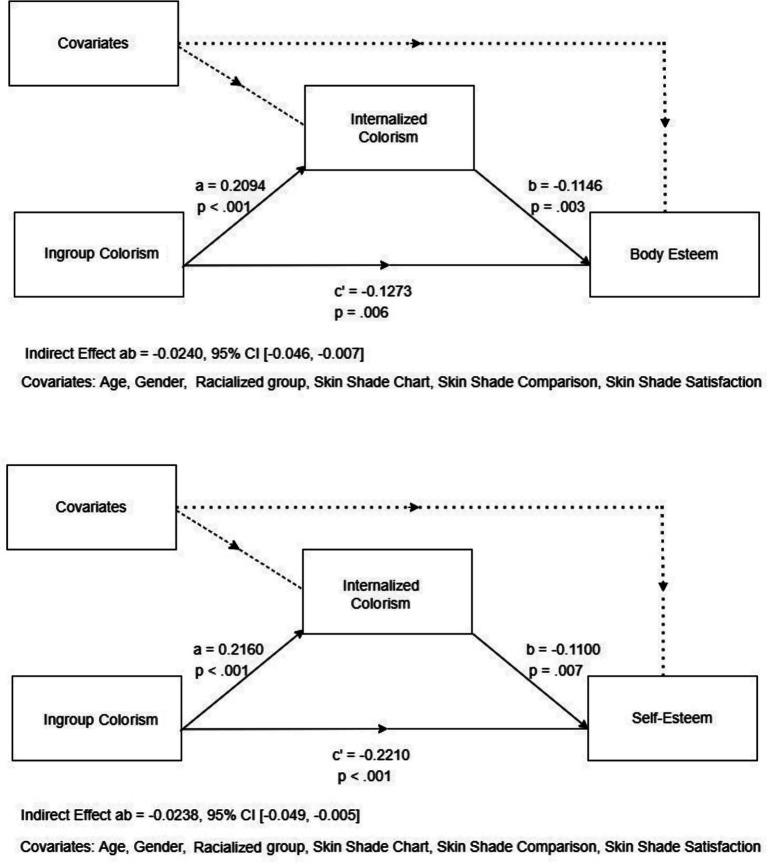
Mediation models for body esteem (top) and self-esteem (bottom).

We also found partial support for our fourth hypothesis (H4). Internalized colorism significantly mediated the relationship between ingroup colorism with both body esteem and self-esteem, with statistically significant indirect effects (*p* < .001 and *p* < .001 respectively) and with statistically significant remaining direct effects (*p* = .006 and *p* < .001) indicating partial mediation. See Figure 2.

In contrast, outgroup colorism showed no significant mediation via internalized colorism for either body esteem or self-esteem. Although outgroup colorism significantly predicted internalized colorism (*p* = .045 and *p* = .026, respectively), the indirect effects were not statistically significant (*p* = .083 and *p* = .052 respectively) and the direct effects, although significant for body esteem (*p* = .009) were not for self-esteem (*p* = .070). These findings suggest that internalized colorism is a key mechanism linking ingroup colorism to body esteem and self-esteem.

Notably, age and racialized group were significant in both models, and gender was significant for body esteem. Specifically, age was inversely associated such that older participants reported lower body esteem and self-esteem. Identifying as Black was associated with higher body esteem and self-esteem. Identifying as a girl was associated with lower body esteem.

## 4 Discussion

The present study examined the associations between experiences of colorism and two key indicators of adolescent psychological wellbeing – body esteem (a dimension of body image) and self-esteem – among Black and South Asian adolescents in the UK. Findings extend research focused on racism and adolescent wellbeing (see Priest et al., 2021) by showing how adolescents’ experiences with colorism are associated with lower body esteem and self-esteem scores. Results revealed nuances based on the source of colorism, highlighting differences depending on whether colorism was experienced by people belonging to the same racialized group as participants (ingroup) or by White people (outgroup). For example, results showed that the relationships between ingroup colorism and both body esteem and self-esteem can be partially explained by the extent to which young people internalize colorist ideas. This was not observed when looking at outgroup colorism. Together, these findings contribute to the growing literature on the psychological associations of colorism (e.g., Adams et al., 2020; Craddock et al., 2023a; Craddock et al., 2023b; Sharif and Siddique, 2020).

Descriptive statistics showed that experiences of both ingroup and outgroup colorism occurred relatively infrequently in the everyday lives of Black and South Asian adolescents. However just 18% of participants reported never experiencing any ingroup colorism and 11% reported never experiencing any colorism from White people, suggesting that most young people in the study have some personal experience with the prejudice. Consistent with our first and second hypotheses, adolescents who reported more frequent experiences of colorism, whether from members of their own racialized group or from White people, also reported significantly lower body esteem and lower self-esteem. Consequently, findings suggest that even relatively infrequent experiences of colorist prejudice may be significant in relation to psychological outcomes. It is possible that colorism’s potency lies not solely in its frequency but in its meaning and the contexts in which it occurs, with certain incidents carrying disproportionate weight.

With respect to our third hypothesis, we anticipated that ingroup colorism would have a stronger impact than outgroup colorism on both body image and self-esteem. This was partially supported. In line with expectations, ingroup colorism emerged as the stronger predictor of self-esteem, consistent with the view that discrimination from within one’s own racialized group may undermine adolescents’ sense of belonging and self-acceptance. It is plausible that when colorism is experienced from trusted ingroup sources such as family members or close peers, it carries unique psychological costs due to its violation of expected solidarity (cf. Drake et al., 2024). However, for body esteem, ingroup and outgroup colorism accounted for similar variance, suggesting that experiences of colorism from racialized minority peers and from White people may be equally salient to adolescents’ body image. Given that societal appearance ideals are largely shaped by the dominant group (i.e., White people), and that colorism is embedded within these ideals (e.g., Hunter, 2021), adolescents’ body image may be particularly sensitive to colorist messages from White people as well as from Black/South Asian people.

Our fourth hypothesis was also partially supported. We found that internalization of colorist beliefs mediated the associations between ingroup colorism and both body image and self-esteem. That is, more frequent exposure to colorism from people belonging to the same racialized background is associated with more negative body image and lower self-esteem in adolescents, and this can be partially explained by the internalization of colorist beliefs. This finding is consistent with qualitative research conducted by Abrams et al. (2020) who found that Black adolescent girls in the USA linked light skin to perceived attractiveness, higher social status, and favorable personality traits, implying a degree of internalization.

In contrast, we found that experiences of colorism from White people were not mediated by internalized colorism, indicating that young people may respond differently to colorism based on the source of colorist experiences. Colorism from White people may be more readily construed as an external manifestation of racism (i.e., a threat or devaluation imposed from an outgroup) and so is not associated with internalization of the prejudice in the same way as when colorism is experienced from the ingroup. Despite this, our data revealed a direct effect between colorism by White people and worse body image, indicating that such outgroup experiences influence body image even in the absence of explicit internalization of colorist ideology. Thus, while internalization helps to explain how ingroup colorism undermines body image and self-esteem, it does not fully capture how outgroup colorism exerts influence. Based on previous research with adults, it is plausible that skin shade surveillance (i.e., the ongoing monitoring and comparison of one’s own skin shade) is a relevant mechanism between colorism from the dominant group (in this case White people) and psychological outcomes. In a UK sample of Black, Asian, and other racialized adults, Craddock et al. (2023a) and Craddock et al. (2023b) found that outgroup colorism was associated with increased skin shade surveillance, which in turn was linked with worse body image.

As colorism is often described as a gendered phenomenon (Hunter, 2021), we examined patterns in the data by gender. Our exploratory analysis showed that both Black and South Asian girls reported higher ingroup colorism than their male peers, suggesting that girls may be particularly vulnerable to ingroup prejudice and mistreatment based on their skin shade. In contrast, there were no differences by gender when comparing colorism from White people among both Black and South Asian participants indicating that outgroup colorism may be experienced more uniformly across girls and boys. These findings align with research conducted with minoritized ethnic adults which showed women reported higher overt experiences of ingroup colorism than men (Craddock et al., 2023b). Additionally, results from this study found that gender was a significant predictor of adolescent body image but not self-esteem. Specifically, being a girl remained significantly associated with lower body esteem in the model that included experiences of colorism, internalized colorism, skin shade, skin shade satisfaction, racialized group and age. This reflects the broader body image literature that finds that adolescent girls often experience more intense appearance pressure than boys and consequently are typically considered to be at higher risk of experiencing poor body image (Calogero and Thompson, 2010).

Interestingly, we found racialized group differences for outgroup but not for ingroup colorism. Both Black girls and boys reported more frequent colorism from White people than South Asian girls and boys, respectively. This is partially consistent with earlier adult data which found that Black participants reported more frequent experiences of colorism from White people and from racialized peers when compared with Asian participants (Craddock et al., 2023b). Racialized group was significant in both linear models indicating that being Black was associated with higher body esteem and self-esteem when controlling for all other variables including experiences of colorism, internalized colorism, skin shade and skin shade satisfaction. This suggests a greater degree of resilience among Black participants, although further research is warranted to examine this more closely, as it is equally possible that other factors are at play (e.g., greater social support).

Our exploratory findings also point to important developmental considerations. Older adolescents in the sample reported more frequent experiences of both ingroup and outgroup colorism. This could reflect growing cognitive maturity and critical consciousness, but it might also indicate that social networks expand in ways that expose adolescents to more diverse and potentially discriminatory interactions over time. In addition, older adolescents may also experience the impact of colorism more acutely as dating and romantic relationships become more salient domains for appearance-related evaluation (Phoenix and Craddock, 2024b). Future longitudinal research could help disentangle these possibilities and examine how the timing and accumulation of colorist experiences shape trajectories of body image and self-esteem.

Finally, our finding that adolescents in this sample were generally satisfied with their skin shade with 85% reporting satisfaction or high satisfaction, is noteworthy. While positive self-evaluations of skin shade were common, these attitudes did not necessarily insulate against the negative impact of colorist experiences on body image and self-esteem. Indeed, both ingroup and outgroup colorism were negatively correlated with skin shade satisfaction, and skin shade satisfaction was strongly and consistently linked to body esteem and self-esteem across gender and racialized groups. This pattern reinforces previous findings that skin shade satisfaction is associated with other appearance-related outcomes as well as well-being more broadly (Ladd et al., 2022), but that its protective capacity may be compromised in the face of repeated or salient discriminatory experiences.

### 4.1 Limitations and future directions

This study is not without limitations. While the cross-sectional design allows for examination of associations, causal inferences cannot be made. Additionally, this study focused on colorist experiences based solely on skin shade. Yet, many definitions of colorism include phenotypical features beyond skin shade, so some nuances may be overlooked. For example, Mitchell Dove (2021) found that Black girls often encounter damaging stereotypes related to hair texture, where straight hair is deemed “beautiful” while curly, coiled or coarse hair is not. Moreover, Mitchell Dove (2021) highlighted how these stereotypes were linked to experiences of discrimination and argued that hair was relevant to Black girls’ identity and self-esteem. Further, we only used one measure of body image. It would be valuable to explore the role of colorism on other aspects of body image such as body appreciation and body dissatisfaction. Relatedly, measuring mechanisms linked to worse body image such as self-surveillance and internalization of a light skin ideal would provide deeper understanding that would be valuable in intervention development. Finally, we only tested one plausible mechanism to explain relationships between experiences of colorism and body image and self-esteem – the internalization of colorist beliefs. Future studies should examine multiple mechanisms simultaneously to provide a more comprehensive understanding of the pathways linking colorism to wellbeing. For example, in line with previous research, it could be worth including skin shade surveillance or internalization of a White appearance ideal specifically (Craddock et al., 2023a; Harper and Choma, 2019) with UK minoritized ethnic adolescents.

Despite these limitations, this research adds to the limited quantitative evidence on colorism among adolescents in the UK, demonstrating its significant association with both body esteem and self-esteem across two racialized groups. The findings underscore the importance of considering not only an individual’s skin shade, their satisfaction with their skin shade or even the internalization of colorist beliefs, but also the direct, lived experiences of discrimination. Findings also demonstrate the value of disaggregating ingroup and outgroup sources of colorism.

### 4.2 Implications

This study contributes to a more nuanced understanding of how colorism operates in adolescence, with clear implications for research, practice, and policy aimed at promoting equitable and affirming environments for minoritized ethnic young people living in the UK. Findings highlight the need for targeted prevention and education efforts in both school and community settings to address colorism explicitly, rather than subsuming it under broader discussions of racism. Second, given the heightened vulnerability observed among South Asian girls, who were found to have lower self-esteem, body esteem, and skin shade satisfaction than Black girls, interventions should be gender- and culturally responsive, incorporating strategies to promote media literacy, critical reflection on societal appearance ideals, and affirmation of diverse skin shades. Third, the finding that ingroup colorism can be particularly detrimental to self-esteem suggests that family- and peer-based interventions may be especially important. These could include workshops or facilitated dialogues within minoritized ethnic communities to challenge entrenched biases and promote intra-group solidarity and ethnic pride. Notably, Rosario et al. (2021) found that Black girls who actively rejected colorist beliefs reported higher self-esteem, underscoring the connection between appearance-related concerns and the affirmation of Black identity. The same may also apply for South Asian girls.

## 5 Conclusion

This study shows that even infrequent everyday experiences of colorism, whether from one’s own racialized group or from White individuals, is associated with lower body esteem and self-esteem among Black and South Asian adolescents in the UK. With stronger associations between ingroup colorism and body image and self-esteem than outgroup colorism, results indicate a greater psychological cost of prejudice experienced within racialized groups. Further, variations by gender and racialized group suggest the need for more in-depth research focused on specific demographic profiles to better understand cultural and gendered nuances. Recognizing colorism as a distinct form of discrimination, and addressing it explicitly through education, policy, and community initiatives, will be essential to creating more equitable and affirming environments for minoritized ethnic youth.

## Data Availability

The cleaned data supporting the conclusions of this article will be made available by the authors, without undue reservation.
